# Influence of the Linking Order of Fragments of HA2 and M2e of the influenza A Virus to Flagellin on the Properties of Recombinant Proteins

**Published:** 2018

**Authors:** L. A. Stepanova, R. Y. Kotlyarov, M. A. Shuklina, E. A. Blochina, M. V. Sergeeva, M. V. Potapchuk, A. A. Kovaleva, N. V. Ravin, L. M. Tsybalova

**Affiliations:** Research Institute of Influenza, Russian Federation Ministry of Health, Prof. Popova Str.15/17, St. Petersburg, 197376, Russia; Institute of Bioengineering, Research Center of Biotechnology of the Russian Academy of Sciences, Leninsky Ave. 33, bldg. 2, Moscow, 119071, Russia

**Keywords:** influenza, vaccine, HA2, M2e, recombinant protein, flagellin

## Abstract

The ectodomain of the M2 protein (M2e) and the conserved fragment of the second subunit of hemagglutinin (HA2) are promising candidates for broadly protective vaccines. In this paper, we report on the design of chimeric constructs with differing orders of linkage of four tandem copies of M2e and the conserved fragment of HA2 (76–130) from phylogenetic group II influenza A viruses to the C-terminus of flagellin. The 3D-structure of two chimeric proteins showed that interior location of the M2e tandem copies (Flg-4M2e-HA2) provides partial α-helix formation nontypical of native M2e on the virion surface. The C-terminal position of the M2e tandem copies (Flg-HA2-4M2e) largely retained its native M2e conformation. These conformational differences in the structure of the two chimeric proteins were shown to affect their immunogenic properties. Different antibody levels induced by the chimeric proteins were detected. The protein Flg-HA2-4M2e was more immunogenic as compared to Flg-4M2e-HA2, with the former offering full protection to mice against a lethal challenge. We obtained evidence suggesting that the order of linkage of target antigens in a fusion protein may influence the 3D conformation of the chimeric construct, which leads to changes in immunogenicity and protective potency.

## INTRODUCTION

The development a new generation of vaccines capable of providing protection against various influenza A viruses, as well as severe forms of influenza A, for at least 5 years is a global challenge. Influenza A conserved proteins (M2, HA2, M1, NP) have emerged as promising targets for vaccine design. A number of studies that have assessed the highly conserved ectodomain of the M2 protein (M2e) of the influenza A virus as a vaccine antigen have shown potent immunogenicity and efficacy in animals, as well as safety and immunogenicity in humans [1-7]. M2e-based vaccines are not for prophylactic use and do not prevent infection, but they reduce clinical signs by limiting virus replication and offering cross-protection [8-11]. The protection offered by M2ebased vaccines is attributed to antibody production [8, 10, 12, 13]. The mechanism of M2e-induced immunity is mediated by antibody-dependent cellular cytotoxicity and antibody-dependent cell mediated phagocytosis. In contrast to anti-HA antibodies, anti-M2e-antibodies do not prevent virus infection and are not neutralizing, but they can eliminate infected cells by an antibodydependent cellular cytoxicity mechanism and thus reduce viral replication [9, 11, 14].

Recently, an enormous research effort has been focused on the HA2 subunit conserved within the phylogenetic group that mediates the fusion of cellular and viral membranes in endosomes, resulting in entry of the ribonucleic complex into the cytoplasm [15]. Monoclonal antibodies that react with the epitopes localized in the stem region of HA are cross-reactive and can neutralize influenza viruses within one phylogenetic group [16-22]. There are studies that have been devoted to the search for the most promising epitopes HA2 of influenza A viruses I and II phylogenetic groups (amino acid residues (aa) 38–59, 23–185, 1–172, 76– 103, 35–107). The identification of these sites allowed researchers to design recombinant proteins [23-27]. Animal studies have shown that such proteins elicit both humoral and cytotoxic T-cell mediated responses. Moreover, they protect animals against a lethal challenge from homologous and heterologous influenza A viruses from one phylogenetic group.

However, a vaccine carrying several conserved protein epitopes which induce humoral and T-cell-mediated responses and neutralize a broad range of influenza virus strains would offer a more effective protection.

Flagellin represents an appropriate platform for the development of recombinant vaccines against various pathogens of viral and bacterial origin [2, 28]. The adjuvant effect of flagellin is mediated via the TLR5 signaling pathway in CD11c+ antigen-presenting cells, which explains the increase in the immunogenic potential of antigens fused to flagellin and the ability to enhance the CD4+ T-mediated humoral response [28-31]. The role of flagellin as a vaccine platform and an adjuvant at the same time has been demonstrated in multiple infection models, including influenza [2, 6, 27, 32-34].

In this study, we report on the eventuality of producing a recombinant protein containing conserved epitopes of the M2 and HA proteins fused to the Cterminus of full-length flagellin. We designed two chimeric proteins with differing orders of linkage of four tandem copies of M2e and a conserved fragment of HA2 (76–130) from phylogenetic group II influenza A viruses to the C-terminus of flagellin. We compared the effect of different insertion points of the target antigens into flagellin on the structure, stability, and immunogenicity of the recombinant proteins.

## EXPERIMENTAL SECTION

**Selection of a conserved HA2 region from influenza A virus phylogenetic group II**

A search for amino acid sequences for our analysis was carried out using the GenBank and GISAID databases. In order to construct consensuses, sequences were aligned using the MAFFT server (http://mafft.cbrc.jp/alignment/server/index.html) and using either the FFT-NS-I or FFT-NS-2 algorithm (depending on the number of sequences) [35] and analyzed using the Unipro UGENE v.1.14.0 software [36]. Alignment and sequence analysis were performed using the Vector NTI (v10.0) software (Invitrogen, USA). A search for experimental B-cell and CD4+ T-cell epitopes homologous to HA2 fragments was performed in the Immune Epitope Database [37]. A search for possible CD8+ T-cell epitopes was conducted using the NetCTLpan 1.1 server [38] with default search parameters. Three-dimensional HA structure (4JTV models - 4O5I A/Victoria/06/2011 H3N2 - from the RCSB Protein Data Bank) was visualized using the Chimera (1.9) [39]. Visualization of the three-dimensional structures of recombinant proteins was carried out using Chimera 1.5.3 [39]. For homology modeling of the 3D structure of recombinant proteins on primary sequence we used the open web resource Phyre2 [40].

**Construction of expression vectors**

The pQE30 plasmid (Qiagen) was used to construct vectors for the expression of chimeric proteins with different insertion points of target antigens. The chimeric protein Flg-4M2e-HA2 contains flagellin (Flg) from* Salmonella typhimurium*, carrying at the C-terminus four copies of the M2e peptide (two copies M2e consensus among human influenza viruses A – M2eh and two copies of M2e from A/H5N1 – M2ek) and the HA2 subunit conserved region of influenza A viruses from the second phylogenetic group. In the second chimeric protein Flg-HA2-4M2e, the HA2-fragment was linked first to the C-terminus of flagellin, followed by four M2e copies. The chimeric genes were designed using common genetic engineering techniques. The flagellin gene was amplified from the *S. typhimurium *genome by PCR and cloned. Nucleotide sequences encoding the consensus HA2 sequence (aa 76–130) of influenza A viruses of phylogenic group II and tandem copies of M2e were synthesized *in vitro*. The HA2 expression in *Escherichia coli *cells was codon-optimized. As a result, vectors expressing pQE30_Flg_HA2_4M2e and pQE30_Flg_4M2e_HA2 were prepared.

**Expression and purification of chimeric proteins**

The *E. coli *strain DLT1270 was transformed with the pQE30/Flg-HA2-4M2e and pQE30/Flg-4M2e-HA2 plasmids for chimeric proteins expression. The strains DLT1270 are derived from DH10B [41] containing the lactose repressor gene *lacI *intergrated into the bacterial genome. *E. coli *strains were grown in a LB medium supplemented with ampicillin until the mid-log phase (OD_600_ = 0.4–0.7) at 37°C, followed by induction with IPTG at a concentration of 0.1 mM and culturing for another 4 h at 37°C. The cells were treated with lysozyme. The produced chimeric proteins were purified from lysed cells by metal-affinity chromatography on a Ni-column.

**Electrophoresis and western-blot**

Polyacrylamide gel electrophoresis (PAGE) was run under denaturing conditions according to the Laemmli protocol [42]. Protein samples were mixed with a sample buffer containing beta-mercaptoethanol, boiled for 7 min, and separated on a 8-16% acrylamide gradient gel. The electrophoresis was run at 10-12 mA for 1.5 h. The gel was fixed in 10% acetic acid. Protein bands were visualized by staining with Coomassie G-250 for 18 h. The proteins were electro-transferred onto a nitrocellulose membrane (BioRad, USA) in a transfer buffer (TB) (0.03 M glycine, 0.04 M Tris, 0.037% sodium dodecyl sulfate, 20% ethanol). The transfer was performed in a Bio-Rad Mini Trans-Blot system (BioRad, USA) at a constant current of 200 mA in the cold (+4°C) for 1.5 h. After the transfer, the nitrocellulose membranes were blocked in 3% BSA (bovine serum albumin, Amresco, EU) in phosphate buffered saline (PBS) overnight at room temperature. Protein bands were visualized by incubation with mouse monoclonal anti-M2e antibodies 14C2 (ab5416, Abcam, UK). The membrane was incubated with primary antibodies diluted in PBS with 0.1% Tween 20 (PBS-T) and 3% BSA, followed by a wash in PBS-T. The bound mouse antibodies were then evaluated with peroxidase-labeled secondary antibodies (goat anti-mouse IgG, Abcam, UK) at room temperature for 1 h and incubated in a TMB (tetramethylbenzidine) Immunoblot solution (Invitrogen, USA) for 5 min.

**Mouse immunization**

Female Balb/c mice (16–18 g) were purchased from the Stolbovaya mouse farm at the State Scientific Center of Biomedical Technologies, Russian Academy of Medical Sciences. The mice were housed at the vivarium of the Research Institute of Influenza of the Ministry of Healthcare of the Russian Federation according to their in-house animal care guidelines. The animals were immunized with Flg-4M2e-HA2 or Flg-HA2-4M2e chimeric proteins intranasally (following inhalation anesthesia with 2–3% isoflurane, 30% O_2_, 70% N_2_O) three times at 2 week intervals at a dose of 6 μg/0.1 ml. The control mice were intranasally injected with 0.1 ml PBS.

**Sampling of sera and bronchoalveolar lavage fluids**

Serum samples and bronchoalveolar lavage fluids (BALF) were obtained from five mice of each group 14 days post third immunization following euthanasia in a CO_2_-chamber (Vet Tech Solutions, UK). Serum was harvested after clot formation at 37°C for 30 min. Blood clots were placed on ice for cooling for 1 h and centrifuged at 400 g for 15 min. The obtained serum was aliquoted (30 μl) and frozen at –20°C.

To collect BALF, the sacrificed animals were secured in supine position on the operating table. The ventral skin was incised from the lover jaw along the midline to expose the trachea. The lower part of the exposed trachea was cannulated 3–5 mm deep to assess the lung lumen. The lungs were lavaged twice via the cannula with 1 ml PBS. The collected BALF samples were centrifuged at 400 g for 15 min and the supernatant aliquoted and stored at –20°C.

**Synthetic peptides**

The immunogenicity of the chimeric proteins was evaluated with the following synthetic peptides supplied by the Scientific Production Association “Verta”:

M2ek SLLTEVETP**T**RNEW**E**CRC**S**DSSD (M2e of influenza virus A/Kurgan/05/05 (H5N1)),

M2eh SLLTEVETP**I**RNEW**G**CRC**N**DSSD (consensus M2e sequence in human influenza A viruses). The different amino acid residues are indicated in bold font and underlined.

## ELISA

Antigen-specific IgG and IgA levels in immunized mice were evaluated with ELISA in high adhesion 96-well plates (Greiner, Germany). The plates were coated with M2e-peptides (5 μg/ml) or purified virus A/Aichi/2/68 (H3N2) (2 μg/ml) in PBS (pH 7.2) overnight at 4°C.

The plates were blocked with PBS in 5% FBS (300 μl/well) at room temperature for 1 h, followed by three washes with PBS-T. The plate wells were loaded with 100 μl 2-fold serum dilutions or BALF in blocking buffer and incubated at room temperature for 1 h. Goat polyclonal anti-mouse IgG and IgA peroxidase labeled (Abcam, UK) in a dilution of 1 : 20,000. TMB was used as a substrate (BD Bioscience, USA). The incubation time was 15 min. The optical density (OD) was measured at 450 nm using an iMark microplate reader (Bio- Rad, USA). The maximum serum dilution that had an optical density at least 2 times higher than the twice mean value of the blank was taken as the titer.

**Viruses and challenge of mice**

In this study, we used influenza virus A/Aichi/2/68 (H3N2) received from the Collection of Influenza and Acute Respiratory Viruses of the laboratory of evolution of the influenza virus at the Research Institute of Influenza of the Ministry of Healthcare of the Russian Federation. Influenza virus A/Aichi/2/68 (H3N2) is a mouse-adapted virus obtained by the Research Institute of influenza by serial mouse/egg passages. The mouse-adapted virus A/Aichi/2/68 (H3N2) retains the antigenic properties of the wild-type strain but acquires a lethal phenotype for mice. The amino acid sequence of the surface proteins (M2, NA and HA) of the mouse-adapted strain was identical to that of the parental strain [6]. On day 14 post third immunization, Balb/c mice (eight mice in the experimental and con trol groups) were challenged with the mouse-adapted A/Aichi/2/68 (H3N2) strain at a dose of 5LD_50_. The virus was administered intranasally in a volume of 50 μl/mouse following inhalation anesthesia (2–3% isoflurane, 30% O_2_, 70% N_2_O). The protective effect of chimeric proteins was measured daily by weight loss and survival rates over a post-challenge period. Control mice were used as negative control in challenge studies.

**Influenza virus replication in lungs**

Mice (three from each group) intranasally received the influenza viruses A/Aichi/2/68 (H3N2), A/PR/8/34 (H1N1), and A/Kurgan/5/05 (H5N1) with 5 times the LD_50_ doses (5LD_50_) on day 14 post third immunization. On day 6 post challenge, the mice were euthanatized (in a CO_2_- euthanasia chamber, Vet Tech Solutions) and their lungs aseptically extracted. The lungs were homogenized in 2.7 ml PBS (Tissue Lyser II homogenizer, Qiagen, USA) to obtain a 10% (w/v) suspension, centrifuged at 400 g at 4°C for 15 min to remove cellular debris and stored at –20°C. The MDCK cell culture in the MEM medium grown in 96-well plates was used for virus titration. Culture cells were infected with 10-fold dilutions (10-1 to 10-8) of the lung homogenate in quadruplicates and incubated in a thermostat (36.0 ± 0.5°C) for 72 h. Following incubation, culture suspensions were transferred into the 96-well plates for immunological assays, followed by the addition of an equal volume of 1% chicken erythrocytes in PBS. The viral titers were determined by hemagglutination test with 0.5% chicken erythrocytes. The viral titers were calculated by the Reed and Muench method. A value opposite to the decimal logarithm of the highest virus dilution showing a positive HA reaction was taken as the titer. Virus titers were expressed as a lg 50% tissue culture infectious dose (TCID50).

**Statistics**

The statistical analysis was done by using GraphPad Prizm v6.0. Statistically significant differences in the antibody levels between groups were tested using the nonparametric Mann-Whitney test. Survival rates were compared with the Montel-Cox test. Differences were considered significant at *p* < 0.05.

## RESULTS AND DISCUSSION

**HA2 fragment analysis (76–130) of influenza viruses from the second phylogenetic group**

**Fig. 1:**
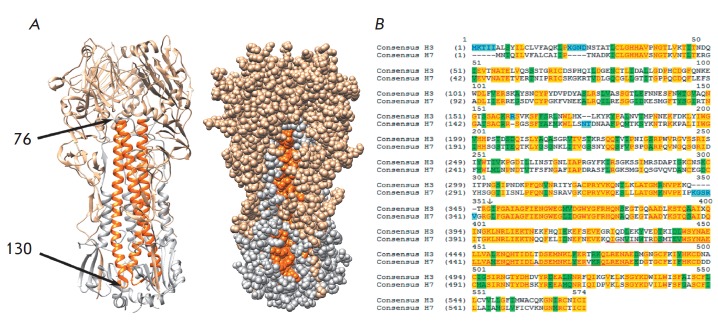
A – Three-dimensional structure (trimer, 4JTV RCSB Protein Data Bank model) of HA of influenza A/H3N2 virus (phylogenetic group II). HA1 subunit is in beige; HA2, in grey; and HA2 fragment 76–130 is in orange. B – Alignment of consensus HA sequences of human influenza A/H3N2 and A/H7N9 influenza viruses (including human isolates), both from phylogenetic group II. The start of the HA2 subunit is marked with an arrow. The 76–130 region is underlined in red, the identical regions are in yellow, substitutions for chemically similar residues are in green, amino acid substitutions are colorless, and insertions are blue.

**Fig. 2:**
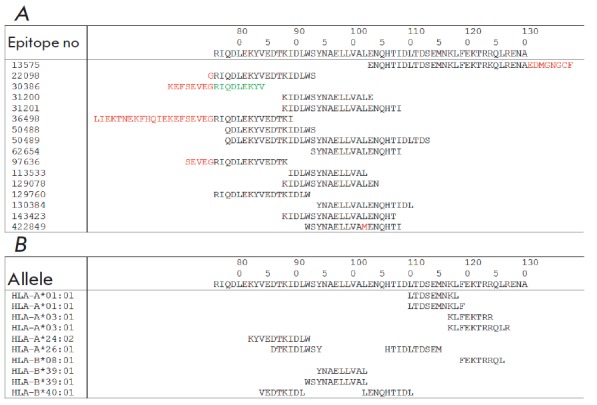
*A *– Experimental *B- *and CD4+ T-cell epitopes that are > 90 % homologous to the consensus HA2 region spanning aa 76–130. The data obtained from the IEDB database. Mismatched amino acids are in red, the B-cell epitope is in green. *B *– Predicted CD8+ T-cell epitopes within the 76–130 region of HA2 for a given set of HLA alleles. Epitopes are predicted by the NetCTLpan1.1 Server [38]

The HA2 fragment (76–130) is a large α-helix in the second subunit of HA partially exposed to the HA surface (*Fig. 1A*). Consensus HA sequences of influenza A viruses from the phylogenetic group II (H3 and H7 subtypes) share 63.6 % identity within the HA2 (76–130) region (*Fig. 1B*). When substitutions of amino acid residues for chemically similar ones are taken into account, the HA sequences exhibit 80% homology. For the influenza viruses of phylogenetic group II, predicted B- and CD4+ T- cell epitopes are located in the first portion of the 76–130 region (*Fig. 2A*). In addition, the HA2(76–130) of influenza viruses of phylogenetic group II contains the predicted CD8+ T- cell epitopes of different HLA alleles (*Fig. 2B*).

**Design of chimeric proteins**

**Fig. 3:**
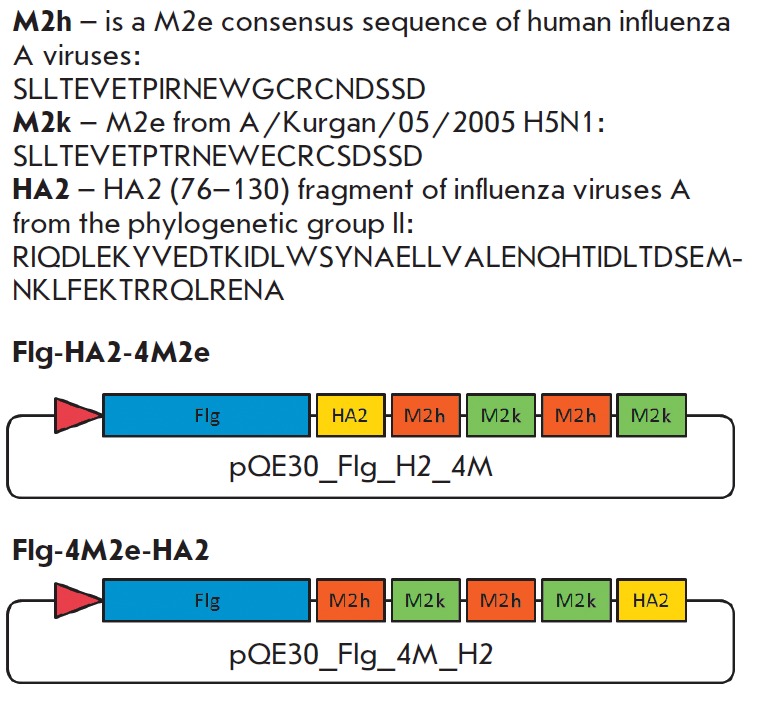
Structure of the chimeric proteins Flg-HA2-4M2e and Flg-4M2e-HA2. The blue block depicts flagellin of* S. typhimurium*; the orange block depicts the consensus M2e sequence of human influenza A viruses; the green block depicts the M2e peptide of influenza A/Kurgan/ 5/05 RG (H5N1) virus; the yellow block depicts the aa 76–130 of HA2 of influenza viruses from phylogenetic group II

The M2eh consensus sequence across human influenza A virus strains, the M2ek sequence of the influenza A/Kurgan/05/200 (H5N1) virus, the HA2 (76–130) fragment of hemagglutinin of phylogenetic group II were selected as conserved peptides to be used in the vaccine design (*Fig. 3*).

We constructed genes expressing chimeric proteins that contain M2e-peptides of different influenza virus subtypes and a conserved fragment of the second HA subunit of influenza A viruses of phylogenetic group II linked at the C-terminus of flagellin in a different sequence order (*Fig. 3*). The chimeric protein Flg-HA2- 4M2e consists of the 76–130 region of the second HA subunit of influenza viruses from phylogenetic group II and four tandem copies of M2e (M2h-M2k-M2h-M2k) sequentially fused to the C-terminus of the flagellin molecule. In fusion protein Flg-4M2e-HA2, four tandem copies of M2e (M2h-M2k-M2h-M2k) were linked to the C-terminus of flagellin, followed by the HA2(76-130) fragment. The M2e copies are separated from each other and from HA2 by glycine-rich linkers. The assembly of the chimeric genes was carried out in the expression vector pQE30. The flagellin gene, without its own start codon, was cloned into the BamHI site of the vector. The expression allowed us to produce recombinant flagellin with the N-terminal 6-His tag needed for purification by metal-affinity chromatography.

The chimeric genes were constructed using common genetic engineering techniques. The flagellin gene was produced by amplification of *S. typhimurium *genomic DNA and cloned. The HA2 nucleotide sequences and M2e tandem copies were generated *in vitro*. Overall, we created pQE30_Flg_HA2-4M2e and pQE30_ Flg_4M2e- HA2 vectors expressing the corresponding proteins.

**Fig. 4:**
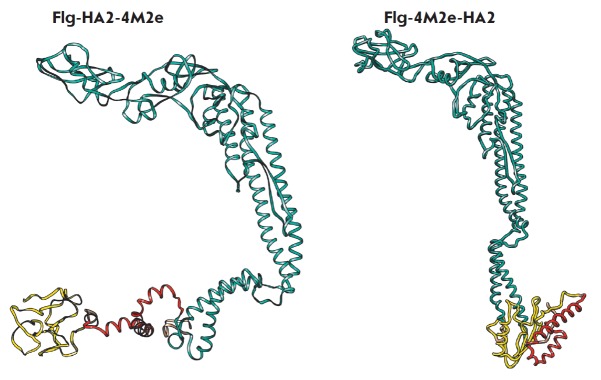
Modeling of 3D- structures of monomeric chimeric proteins Flg- HA2-4M2e and Flg-4M2e-HA2. M2e-peptides are in yellow, HA2 are in red, and Flg is in blue. Protein models are predicted using the Phyre2 server. Structures are visualized with the UCSF Chimera tool

Homology modeling of 3D structures of the Flg-HA2-4M2e and Flg-4M2e-HA2 proteins showed retention of the alpha-helical structure within the 76–130 region of HA2 regardless of the sequence order (*Fig. 4*). It is tempting to say that the native structure of the HA2 fragment seems to remain intact and that the obtained chimeric proteins are immunogenic, including eliciting an antibody response to the structural epitopes occurring in the native HA structure. However, the two chimeric proteins do differ in structure. The chimeric protein carrying the HA2 peptide at the C-terminus displays a more compact structure. The 3D-structure of four M2e tandem copies differed between the two chimeric proteins. When positioned between Flg and HA2 (Flg-4M2e-HA2), the M2e repeats adopted a partial alpha-helical configuration, which does not occur in the native M2e structure on the surface of the virion or infected cells (*Fig. 4*). The terminal position of the M2e tandem copies (Flg-HA2-4M2e) did not significantly alter the intrinsically unstructured M2e conformation existing in the M2 protein. These conformational differences in the structure of the two chimeric proteins may affect their immunogenic properties.

**Production and purification of chimeric proteins**

**Fig. 5:**
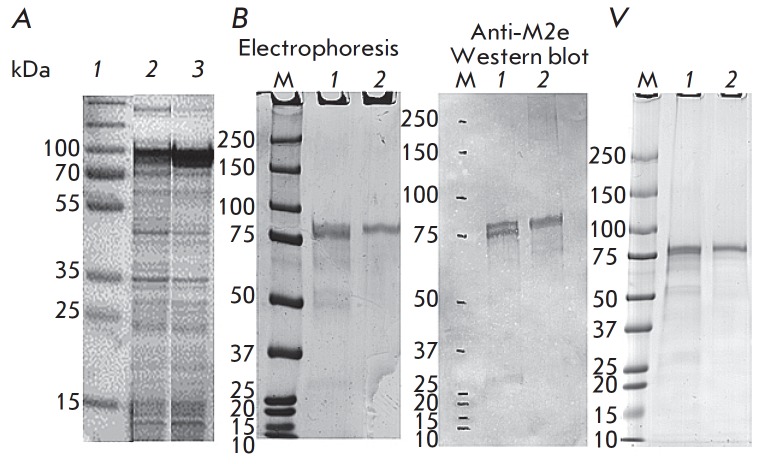
*A *– Expression of the chimeric proteins in *E. coli* cells*. Lane 1, *molecular weight markers, kDa (Fermentas, EU); lane *2*, lysed cells transformed with the pQE30 Flg_HA2_4M2e vector following induction; lane *3*, lysed cells transformed with the pQE30 Flg_4M2e_HA2 vector following induction; *B *– Chimeric proteins Flg-HA2-4M2e and Flg-4M2e-HA2 purified by Ni-affinity chromatography. Electrophoretic profiles and results of western blot analysis using anti-M2e monoclonal antibodies (14C2) are shown; *C ***– **Recombinant proteins Flg-HA2-4M2e and Flg-4M2e-HA2 following a 2-month storage period at 4°C. Electrophoretic profiles are shown: M is a molecular weight marker, *1 *– Flg-HA2-4M2e, *2 *– Flg-4M2e-HA2

The genes encoding the chimeric proteins Flg-4M2e-HA2 and Flg-HA2-4M2e were cloned into the pQE30 vector and expressed in *E. coli *DLT1270 cells (*Fig. 5A*). The expected molecular weight of the two proteins, 73.9 kDa, was in agreement with the molecular weight resolved by electrophoretic migration on PAGE (*Fig. 5B*). The purified proteins Flg-4M2e-HA2 and Flg-HA2-4M2e were recognized by the monoclonal anti-M2e antibodies 14C2 in western blot (*Fig. 5B*). Since 14C2 antibodies recognize only the protective epitope M2e [14, 43], these findings confirm that the M2e peptide is present in both proteins. Both chimeric proteins showed robust stability. When stored at 4°C for 2 months, no sign of degradation was detected (*Fig. 5C*).

**Comparison of the immunogenic properties of the Flg-HA2-4M2e and Flg-4M2e-HA2 proteins**

**Fig. 6:**
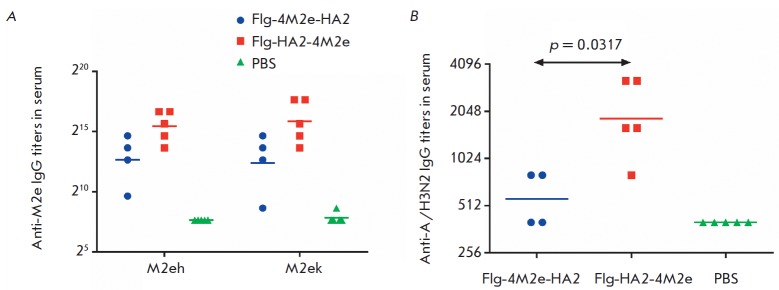
Serum antibody titers in mice of experimental and control groups on day 14 post third immunization with Flg-HA2-4M2e and Flg-4M2e-HA2. Serum IgG levels to the target antigens: A **– **M2e peptides; B **– **influenza virus A/Aichi/2/68 (H3N2)

The immunogenicity of the proteins Flg-HA2-4M2e and Flg-4M2e-HA2 were evaluated in Balb/c mice immunized intranasally three times. On day 14 post third immunization, sera and BALF samples of five mice were tested by ELISA for anti-M2e- and anti- A(H3N2)-antibodies. The mice immunized with Flg-HA2-4M2e or Flg-4M2e-HA2 showed no difference in anti-M2eh and anti-M2ek IgG levels in serum (*p *> 0.05) (*Fig. 6A*). However, the geometric mean titer (GMT) of the anti-M2e IgG levels in mice that had intranasally received Flg-HA2-4M2e was 4- to 6-fold higher than in mice immunized with Flg-4M2e-HA2. The level of anti-HA2 IgG against influenza virus A/Aichi/2/68 (H3N2) was significantly higher in mice immunized with Flg-HA2-4M2e (*Fig. 6B*) (*p *= 0.0317).

**Fig. 7:**
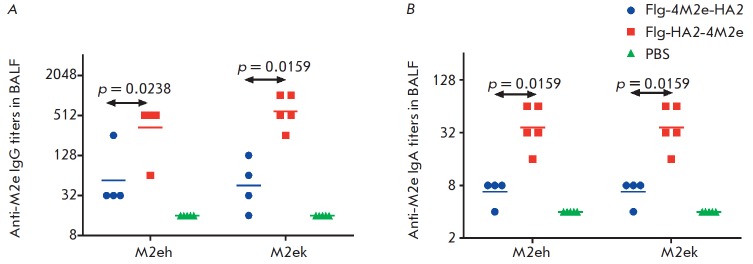
BALF antibody titers to M2e peptides in mice of experimental and control groups on day 14 post third immunization with Flg-HA2-4M2e and Flg-4M2e-HA2: A – IgG; B – IgA

The mucosal IgA and IgG responses to M2eh and M2ek antigens were evaluated in BALF of five mice from each group on day 14 post third immunization. As shown in *Fig. 7*, intranasal immunization with the chimeric protein Flg-HA2-4M2e promoted much higher anti-M2e IgG and IgA levels than Flg-4M2e-HA2 (*p * < 0.05). This result demonstrates that the C-terminal position of M2e in Flg-HA2-4M2e shows more immunogenicity as compared to the inside position of M2e (Flg-4M2e-HA2).

**Comparison of the protective potency of Flg-HA2-4M2e and Flg-4M2e-HA2**

**Fig. 8:**
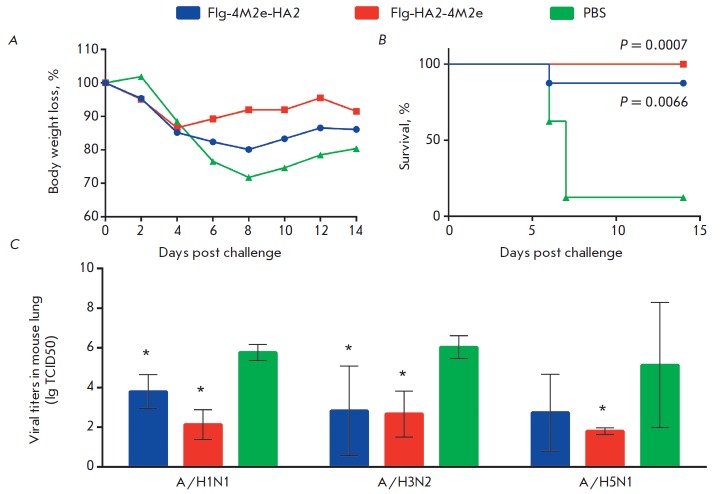
Protective effects of Flg-HA2-4M2e and Flg-4M2e-HA2. On day 14 post third immunization, mice were intranasally challenged with influenza virus A/Aichi/2/68 (H3N2) at a dose of 5LD_50_. Body weight loss (*A*) and survival (*B*) were monitored daily for 14 days. The *p *value was calculated using the Mantel-Cox test. C – viral replication in mouse lung. On day 14 post third immunization, mice were intranasally challenged with influenza viruses A/Aichi/2/68 (H3N2), A/PR/8/34(H1N1), and A/Kurgan/5/05 (H5N1) at 5LD_50_. Viral titers were evaluated on day 6 post challenge. Results are given as lg TCID50. The limit of detection was 0.5 lg TCID50. The statistical significance between experimental and control groups was assessed using the nonparametric Mann-Whitney test. *The differences were considered significant when *p* < 0.05

To evaluate the protective properties of the chimeric proteins, mice (8 mice/group) were immunized with Flg-HA2-4M2e or Flg-4M2e-HA2 and challenged with influenza virus A/Aichi/2/68 (H3N2) with 5LD_50_ on day 14 post third immunization. PBS-inoculated mice that received lethal influenza A/Aichi/2/68 (H3N2) were used as a negative control. The infected mice were monitored daily for body weight changes (as a measure of influenza infection severity) and survival for 14 days. * Figure 8A *demonstrates that mice immunized with the Flg-HA2-4M2e protein showed no more than a 13% body weight loss by day 4 post challenge, whereas mice immunized with the Flg-4M2e-HA2 protein exhibited a 20% decrease in body weight by day 8 post challenge. The maximum body weight loss in the control mice was 28% by day 8 post challenge. These findings show that immunization with Flg-HA2-4M2e offers a milder course of infection compared with the Flg-4M2e-HA2 protein (*Fig. 8A*). Immunization of mice with the fusion protein Flg-HA2-4M2e provided complete protection (*Fig 8 B*.) from a lethal challenge (100% survival), whereas the survival rate of mice treated with Flg-4M2e-HA2 was 87.5%.(p = 0.0007 and p = 0.0066, respectively, Montel- Cox test). The lethal challenge of PBS-inoculated mice resulted in a 12.5% survival rate.

After 14 days post third immunization, all groups (3 mice/group) were intranasally challenged with the influenza viruses A/PR/8/34 (H1N1), A/Aichi/2/68 (H3N2), and A/Kurgan/05/05 (H5N1) with a 5LD_50_ dose. On day 6 post challenge, mice were sacrificed to measure virus titers in their lungs. Mice from both immunized groups had lower viral titers as compared to control mice (*Fig. 8C*). The immunization with Flg-HA2-4M2e led to a 3.7, 3.3, and 3.3 lg reduction in viral titers after challenge with the influenza viruses A/PR/8/34 (H1N1), A/Aichi/2/68 (H3N2), and A/Kurgan/05/05 (H5N1), respectively. These values significantly differed from mock-inoculated mice (*p * < 0.05, Mann-Whitney Test). The chimeric protein Flg-4M2eh-HA2 induced a milder decrease in virus replication levels in the lungs (2.0, 3.2 and 2.4 lg, respectively), although the differences from the control group were significant (*p * < 0.05).

## CONCLUSION

The highly conserved ectodomain of the matrix protein M2 and the conserved regions of the second HA subunit are promising antigens for the development of influenza vaccines with a broad spectrum of protection. The design of a candidate vaccine protein with two or more conserved target antigens that could induce different arms of immune responses (antibodies with different modes of action, CD4+, CD8+ T-lymphocytes) would boost the efficacy of such protein-based vaccines. The recombinant protein based on flagellin and the conserved antigens of two influenza proteins (M2e and aa 76–130 of of HA2) combines the adjuvant activity of flagellin due to TLR5 recognition, a highly conserved structure of M2e between human and avian influenza A virus strains, and a conserved fragment of the second subunit of HA with B-cell, as well as CD4+ and CD8+ T-cell epitopes.

We designed two chimeric proteins based on flagellin varying the insertion points of M2e peptides of different influenza A subtypes and the conserved fragment of the second subunit of HA. The possibility of producing a stable recombinant protein with two targeted antigens (heterologous M2e and HA2 (76–130) fused with flagellin was demonstrated. Such a protein is immunogenic, and it stimulates the formation of antibodies to both M2e and the influenza virus. The recombinant protein protected mice from a lethal challenge and significantly reduced the viral load in their lungs. We found that differing orders of linkage of target antigens to flagellin in the chimeric protein affect the 3D structure of the constructs, its immunogenicity, and protective potency. The two chimeric proteins induced different levels of antibody production, and the Flg-HA2-4M2e protein with a terminus position of M2e peptides was superior to an interior M2e position in the Flg-4M2eh-HA2 protein. Differences in protective effect between the two variants of protein design were also observed. Full protection and rapid recovery of animal weight after a small decline following a lethal challenge was observed in mice immunized with the Flg-HA2-4M2e protein. Moreover, this protein effected a greater reduction of viral titers in the lungs of the animals as compared to Flg-4M2eh-HA2.

Further research would be aimed at clarifying the role of each of the targeted antigens in the fusion protein in the formation of protective immunity, immune response duration, and the duration of its conservation and cross-protective effect.

## Abbreviations

M2e: ectodomain of M2 protein of influenza A viruses
HA: hemagglutinin
HA2: second subunit of hemagglutinin
Flg: flagellin
BALF: bronchoalveolar lavage fluid
ELISA: enzyme-linked immunosorbent assay
OD: optical density
GMT: geometric mean titer
TCID50: 50% tissues cytopathogenic infectious virus dose
